# Enhanced Near-Surface Flaw Detection in Additively Manufactured Metal Ti-5Al-5V-5Mo-3Cr Using the Total Focusing Method

**DOI:** 10.3390/s25206425

**Published:** 2025-10-17

**Authors:** Kate van Herpt, Mohammad E. Bajgholi, P. Ross Underhill, Catalin Mandache, Thomas W. Krause

**Affiliations:** 1 Department of Physics, Engineering Physics and Astronomy, Queen’s University, Kingston, ON K7L 3N6, Canada; 2 Department of Mechanical Engineering, École de Technologie Supérieure (ÉTS), Montréal, QC H3C 1K3, Canada; 3 Department of Physics and Space Science, Royal Military College of Canada, Kingston, ON K7K 7B4, Canada; 4 Aerospace Research Centre, National Research Council Canada, 1200 Montreal Road, Ottawa, ON K1A 0R6, Canada

**Keywords:** non-destructive testing (NDT), total focusing method (TFM), phased array ultrasonic testing (PAUT), additive manufacturing (AM), near-surface flaw detection

## Abstract

Additive manufacturing (AM) enables the fabrication of complex components with high geometric freedom, but it can introduce near-surface flaws due to rapid solidification, resulting in porosity and lack of fusion. In addition, localized melting and steep thermal gradients favor the formation of micro-cracks. Conventional ultrasonic techniques have shortcomings in detecting such flaws because of front-wall interference, further affected by surface roughness and anisotropy. This study evaluates the effectiveness of the Total Focusing Method (TFM), an advanced ultrasonic imaging technique implemented in Full Matrix Capture (FMC), for near-surface flaw detection in Laser Powder Bed Fusion (LPBF) AM components. To assess TFM performance, subsurface side-drilled holes (SDHs) in AM Ti-5Al-5V-5Mo-3Cr (Ti-5553) material were used as the reference reflectors and compared with Phased Array Ultrasonic Testing (PAUT) under identical conditions. Results showed that TFM achieved higher spatial resolution and more reliable detection of shallow flaws, successfully detecting features as shallow as 0.40 ± 0.05 mm below the surface, whereas PAUT was limited to greater depths. These findings demonstrate TFM as a reliable non-destructive evaluation method for shallow flaws in AM parts, while contributing one of the first systematic comparative datasets of PAUT and TFM for shallow SDHs in LPBF titanium alloys.

## 1. Introduction

Additive manufacturing (AM) is an advanced fabrication method for creating three-dimensional (3D) components directly from computer-aided design (CAD) models through the sequential deposition of material layers [1]. Introduced in the 1980s as a rapid prototyping technology, AM has developed into a mature manufacturing platform through sustained advancements in materials science, digital modeling tools, and process control techniques. Today, it enables the production of near-net-shape parts with complex geometries, reduced material usage, and shorter fabrication cycles [2,3]. The ability to produce lightweight, customized, application-specific components—beyond the constraints of subtractive methods—has established AM as a key technology for high-value sectors. As a result, it has been widely adopted in aerospace, biomedical, automotive, and energy industries, where performance, design flexibility, and tailored functionality are paramount [4,5,6].

Despite these advantages, AM is inherently prone to process-induced flaws such as porosity, lack of fusion (LOF), and micro-cracks. Porosity and LOF commonly arise from rapid solidification and incomplete melting or overlap of tracks, while micro cracks are typically associated with localized melting, steep thermal gradients, and the resulting residual stress buildup [7]. These flaws can significantly compromise the fatigue life and structural integrity of AM components. Near-surface flaws are particularly critical, as they often serve as initiation points for crack propagation under cyclic loading. However, the layered build process, anisotropic grain structure, and high surface roughness complicate ultrasonic wave propagation—introducing scattering, attenuation, and distortion—which in turn degrade flaw detectability and sizing accuracy [8,9,10,11].

Ultrasonic Testing (UT) is widely recognized as an effective non-destructive testing (NDT) method for evaluating additively manufactured components, offering capabilities such as subsurface flaw detection, microstructural characterization, and residual stress measurement [12,13,14]. However, conventional single-element UT techniques often struggle with near-surface flaw resolution due to front-wall echo interference and beam steering. To overcome these challenges, advanced array-based techniques—such as PAUT and near-surface Total Focusing Method (TFM)—have emerged as powerful alternatives [12,13,14].

PAUT uses multiple piezoelectric elements with programmable delays to steer and focus the ultrasonic beam in real time. In contrast, TFM is a post-processing algorithm based on Full Matrix Capture (FMC) data that reconstructs fully focused images across the entire region of interest. In FMC, each element of the array probe is sequentially excited, and the responses at all receiving elements are recorded, resulting in a complete matrix of signals containing comprehensive ultrasonic information about the inspected volume. This data acquisition strategy enables high flexibility in subsequent image reconstruction, yielding high-resolution images with improved contrast and sensitivity for detecting small or challenging indications such as inclusions and porosities [15,16,17].

From a theoretical perspective, TFM reconstructs each pixel in the imaging grid by applying delay laws to the full FMC dataset and coherently summing the resulting signals. This operation is mathematically equivalent to synthetic focusing at every point within the region of interest, enabling lateral resolution close to the diffraction limit. Unlike PAUT, which focuses only at predefined depths or steering angles, TFM exploits the complete transmit-receive aperture and suppresses side-lobe artefacts, thereby delivering higher signal-to-noise ratio (SNR) and superior performance in resolving shallow or complex defects [15,16,17].

These advances reflect a broader trend in ultrasonic imaging, where new insonification strategies and digital processing significantly improve flaw detectability and resolution. Emerging techniques such as Phase Coherence Imaging (PCI) and Plane Wave Imaging (PWI), alongside TFM, offer improved data quality for accurate flaw characterization and sizing [15,18,19,20]. Among them, TFM remains particularly advantageous due to its high probability of detection (POD), amplitude fidelity, and suitability for structural integrity assessment in critical industries [21,22].

Electronic beam steering and dynamic focusing, as enabled by PAUT, show limited performance in resolving shallow flaws due to aperture constraints. In contrast, TFM reconstructs fully focused images by utilizing the complete FMC dataset, significantly enhancing image contrast and flaw localization accuracy—especially at shallow depths. These advantages make TFM an attractive technique for inspecting AM components, where near-surface flaw detection remains a persistent challenge. Detecting such flaws is critical, since they act as stress raisers for crack initiation under cyclic loading and cannot be identified by visual or dye penetrant inspection [10,23,24,25].

Recent studies have applied PAUT and TFM to detect critical defects in AM materials, including internal porosity, LOF, and microstructural heterogeneities. For example, Allam et al. [26] employed the longitudinal-to-longitudinal (L-L) wave mode in TFM to image volumetric LOF clusters in Inconel alloys, but they reported limited resolution for flaws smaller than 200 µm. In a subsequent study, the same group detected isolated porosities at approximately 12 mm depth and as small as 0.5 mm in diameter using the same technique. Furthermore, they demonstrated the use of Lamb surface waves for detecting flaws as shallow as 1 mm in thin-walled components [27].

In a series of investigations, Javadi et al. [28,29,30] explored both PAUT and FMC/TFM techniques for inspecting wire and arc additive manufactured (WAAM) metals (a derivative of Directed Energy Deposition, DED). Their work progressed from evaluating flaw detectability [28], to comparing UT methods [29], and finally to developing and validating a calibration procedure for amplitude-based flaw sizing through destructive testing [30].

While linear array probes are most commonly used in UT of AM parts, alternative configurations have shown enhanced performance. Wang et al. [31] applied TFM using both linear and annular arrays and demonstrated that annular arrays significantly improved 3D focusing and flaw sizing, especially for sub-millimeter defects at a depths of 55 mm. Lamberti et al. [32] evaluated various transducer types and confirmed that annular arrays offer superior flaw detectability. Multiple studies corroborate the higher resolution and accuracy of annular over linear arrays [31,32,33].

The inherent complexity of AM materials—including their anisotropic grain structures, directional attenuation, and rough surfaces—poses persistent challenges for near-surface flaw detection. The layer-by-layer fabrication process, combined with variable processing parameters, introduces multiple acoustic interfaces that increase scattering and signal degradation [11,12,13]. Although efforts by organizations such as the National Aeronautics and Space Administration (NASA) and the Electric Power Research Institute (EPRI) have led to the development of AM-specific calibration blocks, these standards focus mainly on volumetric flaw characterization and have limited applicability for near-surface flaws [32,34,35]. For instance, Koester et al. [35] fabricated UT reference samples with artificial volumetric pores to simulate AM-specific defects, while Lamberti et al. [32] emphasized the need for calibration blocks that reflect acoustic anisotropy, variable attenuation, and realistic part geometries.

More recently, EPRI introduced updated reference standards for AM ultrasonic inspection [36,37], yet these remain focused on deep, volumetric flaws. In this context, side-drilled holes (SDHs) provide a more representative and reproducible benchmark for assessing near-surface flaw detectability in AM components. Other advanced ultrasonic applications, such as PAUT-based detection of intentionally embedded void patterns (e.g., QR-code features in LPBF blocks), further highlight the ability of these methods to resolve fine near-surface discontinuities and artificial flaws in AM materials [38].

Many studies fail to clarify whether TFM algorithms were implemented in-house or by using commercial software. When custom algorithms are used, their methodologies are often poorly documented, limiting reproducibility, hindering cross-study benchmarking and impeding the validation and broader adoption of ultrasonic techniques for shallow flaw detection in AM components.

Various NDT techniques have been explored for flaw detection in AM metals, including radiographic testing (RT), penetrant testing (PT), magnetic particle testing (MT), UT, and eddy current testing (ET) [39,40,41,42]. Among these, UT and ET are particularly complementary: UT provides high sensitivity for internal flaws, whereas ET is highly effective for detecting surface and near-surface flaws in conductive, non-ferromagnetic materials. Halliday et al. [43] developed a transmit-receive eddy current probe, optimized through FEM simulations, capable of detecting sub-millimeter flaws located 1 mm below the surface of AM Ti-5Al-5V-5Mo-3Cr (Ti-5553) components, achieving an 80% detection efficiency despite challenges related to surface roughness and edge proximity.

In the context of NDT, sensor performance—particularly sensitivity, resolution, and signal-to-noise ratio (SNR)—is critical for reliable flaw detection. Techniques such as TFM based on FMC and optimized ET probes improve sensitivity and imaging resolution, enhancing the inspection of AM components [43]. Recent reviews emphasize the pressing need for robust NDT strategies tailored to AM, particularly for identifying internal and near-surface flaws. Approaches like TFM and Eddy Current Array (ECA) offer high-resolution imaging, superior sensitivity, and adaptability for anisotropic microstructures and curved surfaces. These advancements support the development of more standardized, reliable, and application-specific inspection protocols for AM components. Despite the growing interest in AM NDT, most current UT research remains focused on volumetric flaw detection, with near-surface flaws receiving comparatively limited attention [11,13].

To fully integrate advanced ultrasonic techniques into industrial quality assurance workflows, it is essential to establish flaw detectability criteria specific to each AM process. This step is foundational for developing reliable reference standards and inspection procedures that meet regulatory and performance requirements for AM components.

Previous studies have demonstrated the feasibility of TFM for additive manufacturing inspection, but typically under different conditions and flaw types. Most investigations have focused on volumetric porosity or intentionally embedded reflectors, providing valuable insights but without addressing shallow-flaw detection in titanium alloys. In contrast, the present work systematically evaluates TFM against PAUT for side-drilled holes at depths of 0.4–2.1 mm in LPBF titanium components. This inspection regime is particularly challenging due to the combined effects of surface roughness, anisotropy, and front-wall interference, and has not been explicitly evaluated in earlier comparative studies [26,27,28,29,30,37].

To address these limitations, this study evaluates the effectiveness of TFM for detecting and sizing shallow flaws in Laser Powder Bed Fusion (LPBF)—manufactured titanium alloy components in the near-surface area. Side-drilled holes embedded at depths ranging from 0.40 mm to 2.15 mm were used to assess and compare the performance of TFM and PAUT in terms of detection capability, image quality, and sizing accuracy. Linear array probes were selected because they are the most widely available and commonly adopted in industrial applications, making the findings directly relevant to practice. In this study, the term near-surface refers to flaws located within approximately 0–2.5 mm of the entry surface, where detectability is strongly influenced by front-wall echoes, surface roughness, and anisotropy. Near-surface flaws are particularly critical because they act as initiation sites for fatigue cracking; the closer a flaw is to the surface, the higher the local stress concentration factor and the greater the risk of premature fatigue failure.

The novelty of this work is twofold. First, it presents one of the earliest systematic investigations of PAUT and TFM for near-surface flaw detection in LPBF-manufactured titanium components, specifically Ti-5553 using shallow side-drilled holes to establish a reproducible benchmark dataset for assessing flaw detectability in additively manufactured materials. This work addresses a critical inspection depth range that has been largely overlooked in previous ultrasonic studies on AM metals. Beyond this comparative framework, the study introduces an innovative application scenario by adapting TFM for the inspection of near-surface flaws in AM titanium. Second, a key innovation lies in the in-house MATLAB R2022b implementation of the TFM algorithm, which enables full control over essential imaging parameters for experimental optimization and reproducible analysis. Together, these contributions extend TFM from a laboratory imaging tool to a practical qualification framework for additive manufacturing inspection procedures, bridging a critical gap between experimental validation and industrial application.

The remainder of this paper is organized as follows: Section 2 describes the materials and experimental methodology; Section 3 presents the results; Section 4 discusses the findings; and Section 5 concludes the study and highlights the industry implications.

## 2. Materials and Experimental Methodology

This section describes the preparation of the AM reference sample, ultrasonic inspection setup, scanning methodology, and data processing procedures used to evaluate the effectiveness of Total Focusing Method (TFM) for near-surface flaw detection.

### 2.1. AM Sample Preparation

A Laser Powder Bed Fusion (LPBF) AM Ti-5553 reference block was employed in this study to evaluate the detectability of near-surface flaws using ultrasonic inspection techniques. The block was fabricated by the Multi-Scale Additive Manufacturing Laboratory at the University of Waterloo (Ontario, Canada) and was provided by Safran Landing Systems Inc. (Singapore). With dimensions of 30 mm × 30 mm × 190 mm, the block features two sets of five side-drilled holes, introduced post-manufacture to serve as artificial reflectors. Each SDH was drilled with a 0.67 ± 0.05 mm diameter to a nominal length of approximately 8 mm. For the present study, only one set of SDHs was used in the analysis. The entry depths from the inspection surface, measured to the center of each SDH, ranged from 0.40 mm to 2.15 ± 0.05 mm as shown in Table 1.

**Figure 1:**
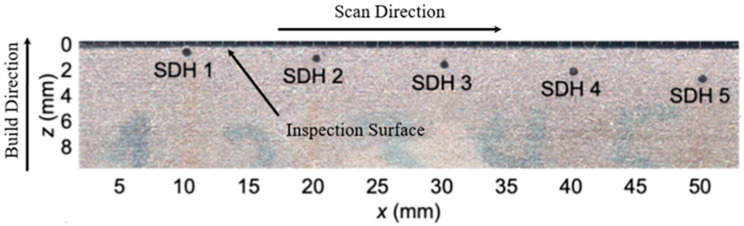
Scaled photographs and geometry of the reference block fabricated via LPBF, including SDHs layout and scan direction used for ultrasonic inspection.

The build direction corresponds to the z-axis, and all ultrasonic inspections were performed on the x–z plane, specifically at the z = 0 mm surface, as shown in Figure 1. This stepped configuration of shallow reflectors follows recognized standards for ultrasonic testing (UT) calibration for near-surface flaw detection [44]. The longitudinal wave velocity along the build direction (z-axis) was measured as (5.42 ± 0.01) × 10^3^ m s^−1^, consistent with reported values for similar titanium alloys [45,46]. The variance in surface roughness of the block was previously reported to be 80 µm^2^ [43].

### 2.2. Ultrasonic Inspection System

**Figure 2:**
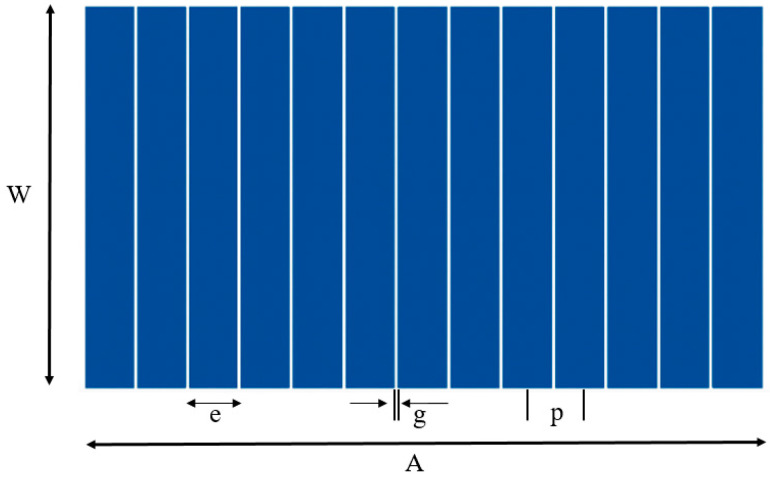
Schematic of the linear phased array probe layout with dimensional parameters.

**Figure 3:**
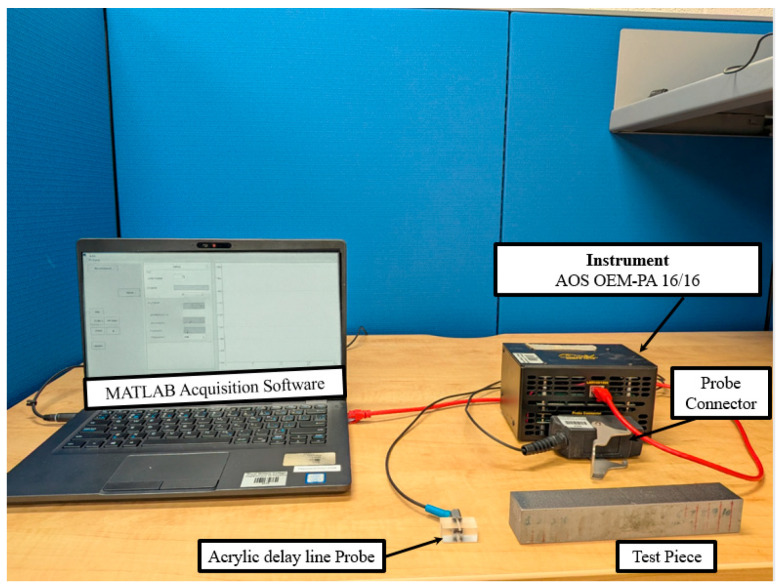
Experimental setup used in this study.

Ultrasonic testing was carried out using the OEM PA 16/16 phased array acquisition system from Advanced OEM Solutions (AOS), West Chester, Ohio, USA, paired with a 16-element linear phased array transducer (Model 00 011207 IPEX) from Sensor Networks, Inc., State College, PA, USA. The transducer operates at a center frequency of 10 MHz, chosen to ensure high spatial resolution since the corresponding wavelength (λ ≈ 0.54 mm) is smaller than the diameter of the SDHs. A schematic of the probe layout is shown in Figure 2, illustrating the dimensional parameters of a linear phased array probe and highlights the main features, including element width (e), pitch (p), kerf (g), active aperture (A), and passive aperture (W) [47]. The key specifications of the ultrasonic phased array transducer employed in this study are summarized in Table 2. The overall experimental setup, including the acquisition system, transducer, and MATLAB acquisition software, is shown in Figure 3.

### 2.3. Data Acquisition Settings

In the current study, data acquisition was performed out using an OEM-PA system configured to excite the 10 MHz phased array transducer with a 50 ns square wave pulse (equivalent to half the cycle). The A-scan signals were digitized at a sampling frequency of 100 MHz with an 8-bit resolution, which was sufficient for reliable flaw detection in this setup. Each inspection scan was repeated 20 times to ensure repeatability, and all data were stored in MATLAB MAT-files (*.mat). The delay laws for both PAUT and FMC modes were sequentially uploaded to the OEM-PA. Initially, acquisitions were conducted in flaw-free regions to establish reference signals. Subsequently, the transducer was manually positioned over the center of each SDH, and both PAUT and FMC data were captured at each flaw location and inspection angle. This stepwise procedure ensured consistent signal quality and allowed for comprehensive comparison across different imaging modalities.

### 2.4. Scan Plan

A scan plan was essential to ensure standardized, repeatable ultrasonic inspections with complete volume coverage [47]. It defined key parameters such as imaging modes, grid density, probe positioning, and scan-line distribution relative to a fixed reference point. This approach enhanced consistency and reliability in advanced techniques like FMC. In this study, six ultrasonic scan configurations were developed to evaluate the detection, localization, and sizing capabilities of SDHs using PAUT and FMC/TFM techniques.

**Figure 4:**
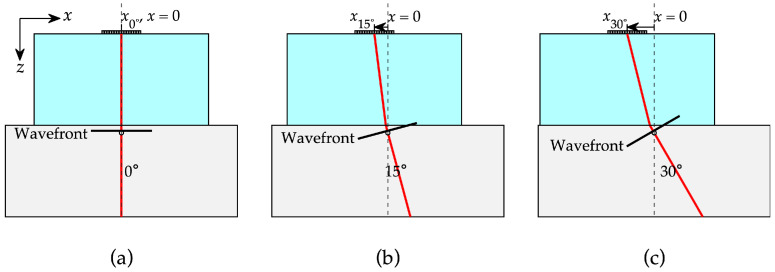
Schematic of the three probe positions with a ray emerging from the center of the array to experience specular reflection from a side-drilled hole (SDH) at (**a**) 0°, (**b**) 15°, and (**c**) 30° in the reference block.

Each PAUT scan consisted of a set of acquisitions defined by an active aperture and associated delay laws for a specific steering angle and/or focal length. Three sectorial scans were performed at probe positions x_0_, x_15_, and x_30_ (corresponding to central steering angles of 0°, 15°, and 30°), as shown in Figure 4. Each scan used 30° sweeps at 0.5° increments, producing 61 unique S-scan acquisitions per scan with a full 16-element aperture and a focal depth of 1 mm. These probe positions were chosen to optimize specular reflection alignment with the SDHs and to reduce interference from front-wall echoes caused by the wedge-block interface.

The remaining three FMC/TFM scans were performed at the same probe positions as the PAUT scans, with FMC acquisition followed by TFM image reconstruction in MATLAB R2022b using the longitudinal-longitudinal (L-L) mode and a grid resolution of λ/20 mm. This scan plan provided a robust framework for comparing the performance of PAUT and TFM in flaw characterization and near-surface imaging.

### 2.5. Data Processing

The data acquisition process in this study was facilitated through the use of an OEM phased array ultrasonic testing (PAUT) system provided by AOS. The system offered core device functionality and an integrated focal law calculator essential for PAUT applications. Capitalizing on the OEM-PA platform’s semi-open-source architecture, the existing software infrastructure was extended and adapted to meet the specific needs for inspection and imaging workflows.

**Figure 5:**
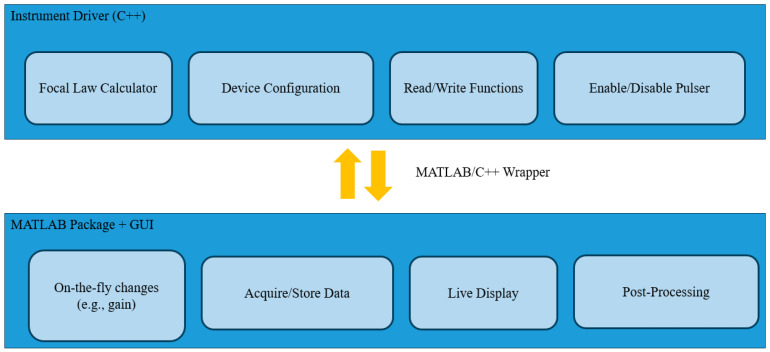
In-house MATLAB implementation of the TFM algorithm providing full control over imaging parameters such as velocity, aperture, and grid resolution.

Software development was carried out using a MATLAB/C++ wrapper supplied by AOS, which enabled low-level control over hardware operations and direct interaction with the data acquisition components. Building on this foundation, a custom MATLAB-based graphical user interface (GUI) was developed to manage the acquisition process. The GUI allowed streamlined control over system parameters, real-time signal visualization, and execution of FMC across varying probe configurations. The in-house implementation of the TFM algorithm developed in MATLAB is illustrated in Figure 5.

A key innovation in this work was the in-house implementation of the TFM algorithm in MATLAB. At the time of testing, TFM capabilities were not available in the version of the OEM software. As a result, a complete TFM processing pipeline was developed from the ground up, providing full control over algorithmic parameters such as sound velocity, aperture size, and reconstruction grid resolution. This flexibility improved transparency and enabled future expansions, including integration of flaw characterization modules and adaptive imaging enhancements. MATLAB was selected as the primary development environment due to its high-level programming capabilities, extensive signal processing libraries, and suitability for rapid prototyping.

The in-house MATLAB implementation provided full control over acquisition and reconstruction parameters, including aperture selection, grid resolution, velocity calibration, and delay-law computation. The OEM-PA communicated with a laptop via Ethernet through manufacturer-supplied C++ drivers and dynamic-link libraries (DLLs). These were expanded in five steps: (1) reviewing and documenting the header files, (2) testing undocumented functions, (3) extending the MATLAB interface for advanced hardware control, (4) implementing routines for device initialization, gain configuration, and delay-law uploading, and (5) designing an intuitive graphical user interface (GUI) for real-time acquisition, visualization, and data storage. The TFM algorithm calculated two-way travel times between each transmit-receive element pair and every imaging pixel, coherently summing the time-aligned signals to reconstruct amplitude maps. This flexible architecture also enabled parameter-sensitivity analysis by allowing controlled variation in aperture width, grid density, and delay-line geometry.

This modular and customizable acquisition framework enabled precise tailoring of the imaging process to meet experimental needs, laying the groundwork for enhanced control, deeper analysis and adaptability in future ultrasonic inspection studies. To enhance reproducibility and provide a concise overview of the experimental instrumentation, the key components used in this study are summarized in Table 3.

## 3. Results

This section presents a detailed analysis of the key factors affecting the performance of TFM imaging in ultrasonic testing. Drawing on experimental findings and critical evaluations of current practices, several parameters are identified as particularly influential: amplitude fidelity, wave mode selection, and flaw detectability and sizing. In addition, a brief sensitivity analysis is presented at the end of Section 3.2 to quantify how grid spacing, aperture, delay-line configuration, and frequency affect TFM image quality and flaw detectability. The following subsections examine each parameter in detail, with a particular focus on TFM’s near-surface detection capability and flaw sizing accuracy.

### 3.1. Amplitude Fidelity in Total Focusing Method (TFM) Imaging

Amplitude fidelity refers to the accuracy with which a TFM image reproduces the true amplitude of ultrasonic signals reflected from flaws within a material. High amplitude fidelity ensures that the relative signal strengths captured in the image correspond closely to the actual flaw responses, which is critical for reliable flaw sizing and characterization. According to ASME Section V [17], when amplitude-based sizing is performed, the amplitude fidelity deviation must not exceed 2 dB, whereas if amplitude is not directly used for sizing, a deviation of up to 4 dB may be tolerated. Poor amplitude fidelity can result in under- or overestimation of flaw dimensions, reduced signal sharpness, and potentially non-compliant inspections [17].

**Figure 6:**
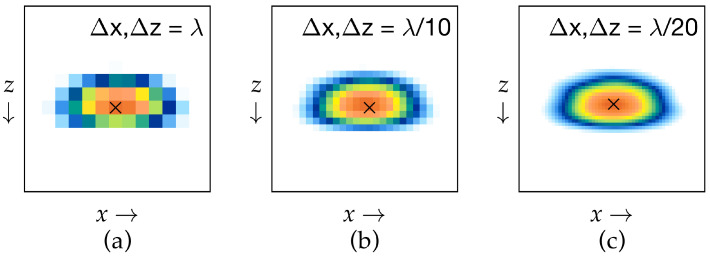
Example of amplitude fidelity preservation achieved at different grid point sizes for SDH 5: (**a**) Δx = Δz = λ, (**b**) Δx = Δz = λ/10, and (**c**) Δx = Δz = λ/20. Colors indicate TFM image amplitude, where red represents the highest echo intensity and blue the lowest.

To maintain amplitude fidelity in the TFM implementation, spatial sampling criteria were applied based on the analytical model proposed by Badeau et al. [48], which defines the relationship between pixel size, wavelength, and amplitude deviation. This model recommends using a pixel size no larger than λ/5 to limit amplitude error to within 2 dB. In the MATLAB-based TFM reconstruction, a finer spatial resolution of λ/20 (Figure 6c)—significantly smaller than the recommended limit—was adopted as part of a targeted grid-spacing optimization, ensuring minimal interpolation error and improved signal representation. This is illustrated in Figure 6, where SDH 5 was reconstructed at different grid point sizes, demonstrating how a finer grid spacing leads to a more stable maximum amplitude. This high-resolution approach enabled accurate detection of peak amplitudes and well-defined echo boundaries, enhancing overall imaging quality and ensuring compliance with amplitude fidelity requirements. By integrating this advanced analytical criterion, robust and reproducible TFM images were achieved, with high confidence in amplitude-based flaw assessments.

### 3.2. TFM Wave Mode

Wave mode selection plays a critical role in TFM imaging, directly influencing flaw detectability, resolution, and penetration depth [16,17]. In this study, the L-L mode was selected as the primary wave mode for TFM reconstruction as part of a targeted wave-mode optimization. This choice was driven by the high attenuation characteristics of AM Ti-5553 material at the 10 MHz test frequency, preventing reliable back-wall reflections and limiting the feasibility of indirect modes.

Direct wave modes, such as L-L, provide straightforward propagation paths between the transmitter and receiver, reducing multipath scattering and improving image clarity—particularly important in materials with interlayer noise, such as AM metals. Although indirect modes can offer enhanced volumetric coverage and better flaw characterization by incorporating reflections or mode conversions, they were not viable in this study. The signal return from the back wall was insufficient in components with a thickness of 30 mm. While lower-frequency transducers could theoretically enable indirect mode operation, they would compromise the resolution needed to detect very fine pores on the order of tens of microns (≈10 µm), typical of AM porosity, which are often only detectable through attenuation effects.

**Figure 7:**
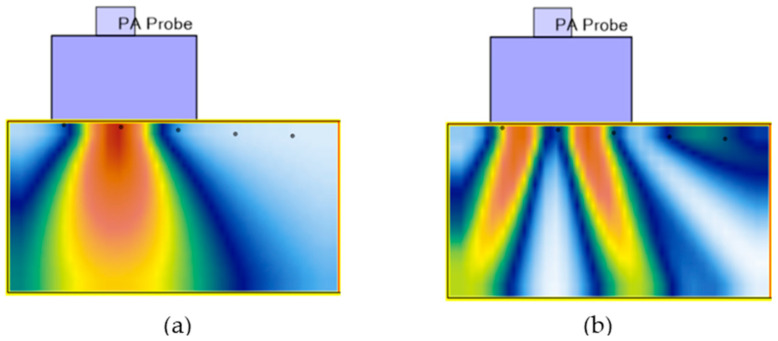
Example of Wave mode selection by Beam Tool 11 software for (**a**) Wave mode (L-L), and (**b**) Wave mode (T-T). Colors represent acoustic pressure amplitude, with red showing the highest intensity and blue the lowest.

Shear waves are often preferred for near-surface flaw detection due to their shorter wavelengths and increased time separation from front-wall echoes. However, their use was not feasible in this study due to the unavailability of appropriate angled wedges to generate the required refracted S-waves. Beam Tool 11 software simulations further confirmed the impracticality of using shear modes under the existing experimental setup (Figure 7). Given these constraints, the L-L wave mode represented the outcome of a balanced optimization, providing the best compromise between penetration depth, resolution, and image reliability for inspecting AM Ti-5553 in this study.

In the current study, a sensitivity analysis was performed to evaluate how variations in TFM parameters influence image quality and flaw detectability. The results confirmed that grid spacing and aperture size exert the strongest control on amplitude stability and flaw boundary sharpness. Finer grid spacing (≈λ/20) improved resolution and reduced amplitude deviation, whereas smaller effective apertures slightly decreased image contrast near the front wall due to reduced focusing energy, as shown in Figure 6. To minimize near-surface interference, a 15 mm-thick acrylic delay line was coupled to the 10 MHz probe. The delay line, made of a low-velocity material, effectively mitigated the transducer dead zone and near-field effects by increasing the time between the initial excitation pulse and the first flaw echo, ensuring that the wave entered the test piece after the ring-down period and thereby improving near-surface detectability. These observations align with amplitude-fidelity criteria in ASME Section V (2023) [17], which specify a 2 dB tolerance when amplitude-based sizing is performed. Preservation of true echo amplitude within these limits depends on grid discretization, material attenuation, probe frequency, wedge geometry, and instrument dynamic range. Wave mode selection remains another dominant factor: the direct L-L mode used here provided a practical balance between penetration depth and near-surface resolution in attenuative AM titanium, while indirect or conversion modes (L-T, T-T, and TT-TT) could enhance volumetric coverage when signal quality allows. Finally, probe and wedge configuration—including aperture width, pitch, and coupling angle strongly influence focusing uniformity and spatial coverage. Collectively, these results highlight that optimizing grid density, aperture design, delay-line geometry, and frequency selection is essential to achieve amplitude fidelity and stable reconstruction, thereby ensuring reliable, high-resolution TFM imaging.

### 3.3. Detection Capability for Near-Surface Flaws

The near-surface detection performance of PAUT and TFM was evaluated using five SDHs (0.67 ± 0.05 mm diameter) embedded at depths ranging from 0.40 to 2.15 mm in an LPBF Ti-5553 block as shown in Table 1.

**Figure 8:**
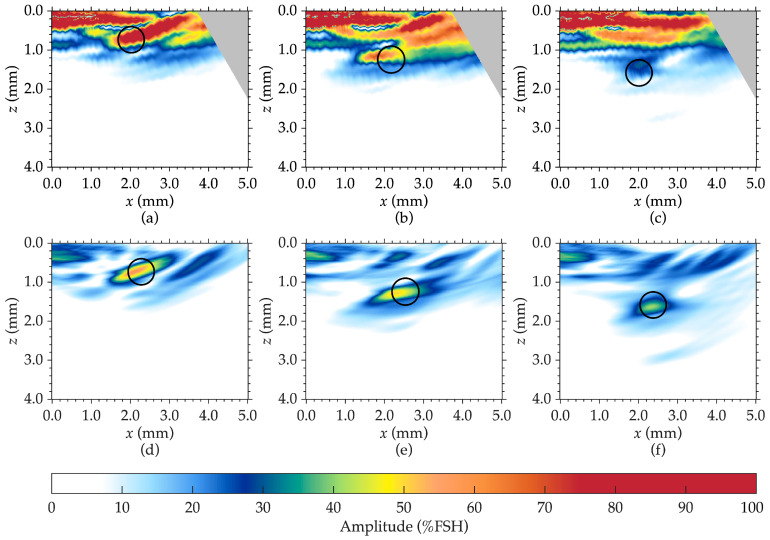
Comparison of PAUT and TFM results for near-surface flaw detection at the x_15°_ probe position, as an example. (**a**) PAUT-SDH 1, (**b**) PAUT-SDH 2, (**c**) PAUT-SDH 3, (**d**) TFM-SDH 1, (**e**) TFM-SDH 2, (**f**) TFM-SDH 3. All images are normalized to the maximum amplitude.

**PAUT performance**: Figure 8a–c shows PAUT results for near-surface flaw detection in additively manufactured metal when the probe is in the x_15°_ position and the indication is centered around the 15° incidence angle for SDH 1, 2, and 3, respectively. At 0°, PAUT reliably resolved flaws at depths ≥1.25 mm (±0.05 mm tolerance), with SDH 2 at 0.90 mm only partially visible and SDH 1 at 0.40 mm fully obscured by the front wall. At 15°, visibility of SDH 2 improved, but SDH 1 remained unresolved. At 30°, SDH 2 was clearly resolved and SDH 1 became faintly visible, though still inseparable from interface echoes. These results indicate a minimum resolvable depth of ~0.90 mm at 30° and ~1.25 mm at lower angles. Broader flaw indications and depth overestimation at higher angles were likely caused by beam divergence and steering-induced distortion.

**TFM performance:** Figure 8d–f shows TFM results for near-surface flaw detection in the additively manufactured metal when the probe is in the x_15°_ position for SDH 1, 2, and 3, respectively. At the x_0°_ position, TFM performance is similar to PAUT, resolving flaws at depths ≥1.25 mm. At the x_15°_ position, TFM achieves a notable improvement, successfully detecting and localizing SDH 1 at 0.40 mm, while resolving all other SDHs. At the x_30°_ position, TFM maintains complete flaw resolution with sharper boundaries and fewer artifacts than PAUT. A minor depth shift at higher angles is attributed to velocity calibration error. TFM’s advantage is linked to reduced front-wall interference from single-element excitation and the ability to synthetically focus on every point in the region of interest. A comparison of PAUT and TFM results in Figure 8 clearly demonstrates that TFM provides superior sensitivity and resolution for identifying small near-surface flaws compared to PAUT, making it the preferred technique for enhanced near-surface flaw characterization in such materials. All PAUT and TFM images were normalized to the reflection amplitude from SDH 5, set to 60% of the dynamic range.

**Comparative trends**: Table 4 summarizes the detection outcomes. Across all angles, TFM consistently produces higher-contrast, more localized flaw images and showed greater robustness to probe position changes. PAUT’s fixed focal laws and concentrated beam produced strong amplitudes but struggles with shallow flaw separation, particularly below 0.90 mm depth. TFM’s fine reconstruction grid and post-processing flexibility enables reliable detection down to 0.40 mm, a range where PAUT provides only partial or ambiguous indications.

### 3.4. Flaw Sizing

#### 3.4.1. Sizing Accuracy

The accuracy of flaw sizing in PAUT is fundamentally limited by beam characteristics—particularly beam width, which restricts lateral resolution. When two indications are closer than the beam width, they may appear as a single signal, leading to an overestimation of flaw size. This is a known limitation of the commonly used 6 dB drop sizing technique [49].

The transducer frequency, which is inversely proportional to wavelength, also plays a key role in determining resolution. Higher frequencies produce narrower beams and improve lateral resolution, theoretically enabling the detection of smaller flaws [47]. However, this comes at the cost of increased attenuation, especially in coarse-grained materials such as additively manufactured metals [8]. In theory, the minimum reliably detectable flaw size is approximately half the wavelength, which, for a 10 MHz probe (wavelength ≈ 0.54 mm), should allow the detection of a 0.67 mm SDH. In practice, however, factors such as beam steering, focus quality, material attenuation, and microstructural noise can significantly influence flaw sizing accuracy.

TFM improves on PAUT by reconstructing an image on a pixel-by-pixel basis using a delay-and-sum algorithm, which amplifies coherent signals and suppresses incoherent noise. Despite this advantage, TFM is also subject to beam divergence, particularly for shallow flaws located farther from the probe. Increased propagation distance leads to spreading of the ultrasonic beam, which can cause flaws to appear wider than their actual size. This effect was observed in both the PAUT and TFM results.

To mitigate this, TFM supports post-processing corrections using a point spread function (PSF), which characterizes how a point reflector appears in the image. Although not implemented in this study, PSF-based deconvolution is a well-established method for improving sizing accuracy by correcting beam spread and reducing apparent flaw width [50]. In the present study, PSF correction was not applied because the primary goal was a relative comparison between PAUT and TFM under identical conditions. Since both techniques were equally affected by beam spreading, the reported improvement with TFM remains valid. Nevertheless, it is emphasized that PSF analysis is essential for future work where absolute flaw sizing accuracy is required, such as in probability of detection studies, fitness-for-service evaluations, and code-compliant qualification of AM parts.

Additionally, flaw sizing in this study was performed in a single plane, perpendicular to the ultrasonic beam. According to ASME BPVC Section V [17], full characterization requires inspection from at least two orthogonal directions. Future improvements could be achieved through the use of shear waves (S-waves), which offer shorter wavelengths and narrower beam diameters than longitudinal waves (L-waves), resulting in enhanced spatial resolution and more precise flaw sizing.

#### 3.4.2. Flaw Sizing in MATLAB

Flaw sizing in this study was carried out using a threshold-based amplitude segmentation technique, where the maximum flaw signal amplitude was identified as the reference and a ±6 dB range was applied to isolate the flaw region while minimizing the influence of background noise. Amplitudes outside this range were discarded, and a binary image was generated by setting irrelevant pixel values to zero.

**Figure 9:**
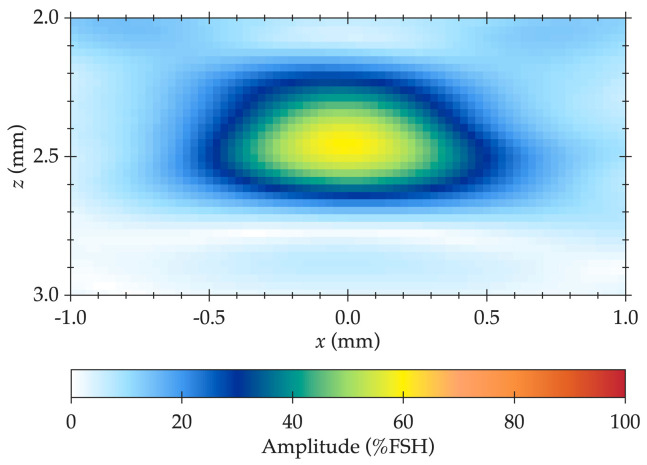
Zoomed-in TFM image of a side-drilled hole in an additively manufactured Ti-5553 block, showing the segmented flaw region used for sizing analysis with the amplitude-drop method in MATLAB.

This binary image was processed using MATLAB’s Image Processing Toolbox. Key geometric features were extracted using the regionprops function [51], including the bounding box enclosing the flaw and the best-fit ellipse outlining its boundary. The major axis (long axis) of the ellipse was taken as the flaw width. This approach provided a consistent and quantitative method for assessing flaw dimensions across multiple acquisitions (Figure 9).

#### 3.4.3. Flaw Sizing Results and Comparison

To evaluate flaw sizing accuracy, an oversizing factor was calculated by comparing the measured flaw width to the true flaw width. Here, W_meas_ denotes the measured width of the SDH using the 6 dB drop technique, and W_0_ represents the true width of the SDH, set at 0.67 mm. The oversizing factor F, defined as

F = W_meas_ ÷ W_0_ (1)

quantifies the deviation of the measured flaw size from the actual flaw size. This metric enables a standardized assessment of sizing accuracy across different ultrasonic imaging methods. The oversizing factors for 0.67 mm diameter SDH widths, as measured using PAUT and TFM, are summarized in Table 5. SDH 1 and 2 were not included in the comparative flaw sizing analysis because their very shallow depths caused their echoes to overlap with the front-wall reflection. While PAUT could not provide reliable sizing under these conditions, TFM enabled limited estimation (Figure 8), but these cases were excluded to maintain consistency in the comparison between the two techniques.

At a depth of 1.25 mm, PAUT produced an oversizing factor of 2.77 ± 0.01, while TFM achieved 2.14 ± 0.01. At depths of 1.75 mm and 2.15 mm, TFM returned oversizing factors of 2.42 ± 0.06 and 2.79 ± 0.04, respectively, compared to 3.35 ± 0.01 and 3.44 ± 0.05 for PAUT. These results demonstrate that TFM provides enhanced flaw sizing performance, particularly in AM Ti-5553 components, where structural heterogeneity and surface roughness pose challenges to conventional UT methods. The uncertainty in each measurement was estimated from the standard deviation of 20 repeated acquisitions per depth. At each depth, the 95% confidence intervals for PAUT and TFM oversizing factors do not overlap, indicating a statistically significant reduction in oversizing with TFM.

These findings—supported by previous literature [11,13,52,53]—confirm that TFM improves flaw sizing reliability in demanding inspection environments. Its superior focusing capability and reduced sensitivity to structural heterogeneity and surface roughness make it a more reliable method for flaw characterization in challenging AM inspection scenarios. These results contribute to the growing body of evidence supporting the adoption of advanced TFM-based imaging in AM inspections and may inform future updates to NDT procedures or standards for flaw sizing in safety-critical additive manufacturing applications.

**Figure 10:**
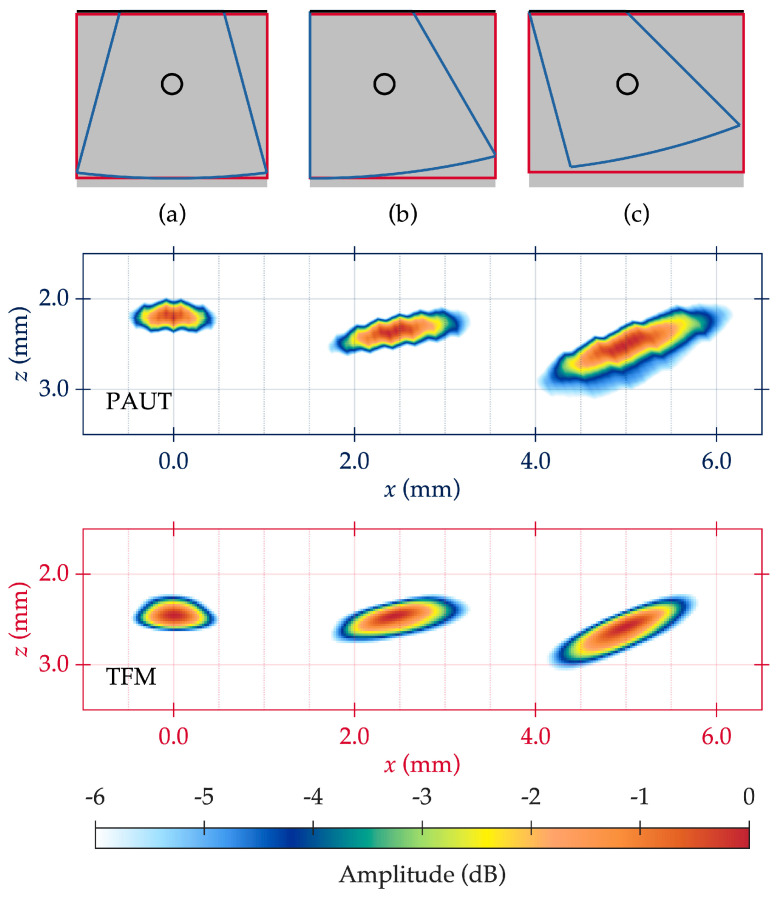
Variation in flaw indication width for a 2.15 mm deep SDH imaged at different probe positions using PAUT and TFM: (**a**) probe position at x_0°_, (**b**) probe position at x_15°_, and (**c**) probe position at x_30°_. Here, the x-axis represents the lateral distance from the probe (in mm), while the z-axis indicates depth (in mm).

To further evaluate the flaw sizing capabilities of PAUT and TFM, an SDH located at a depth of 2.15 mm (SDH 5)—the deepest among the inspected flaws—was imaged from multiple probe positions. All imaging results are shown in Figure 10, which illustrates the variation in flaw indication width as a function of the probe’s lateral distance from the flaw. The top row of Figure 10 shows the imaging geometry: the blue zone represents the PAUT sectorial scan coverage, and the red zone corresponds to the TFM reconstruction area. The leftmost image depicts the probe positioned directly above the flaw at x_0°_, with subsequent images showing the probe gradually shifted laterally to the x_15°_ and x_30°_ positions to increase the distance between the probe and the flaw.

In the middle and bottom rows, the flaw indications captured by PAUT and TFM are presented, respectively. At the central position x_0°_ (directly above the flaw), both PAUT and TFM produced similarly narrow flaw indications, closely matching the true width of the SDH. However, as the probe is moved laterally, the width of the flaw indications increases for both methods due to beam divergence and the longer propagation path. A slight depth overestimation is observed in the TFM images, which is attributed to reconstruction artifacts, particularly the sensitivity of TFM to grid resolution and variations in ultrasonic velocity in additively manufactured alloys. Ensuring accurate velocity calibration is therefore essential, as velocity and frequency dependence in AM materials can directly influence the depth positioning in TFM reconstructions [32,33].

**Figure 11:**
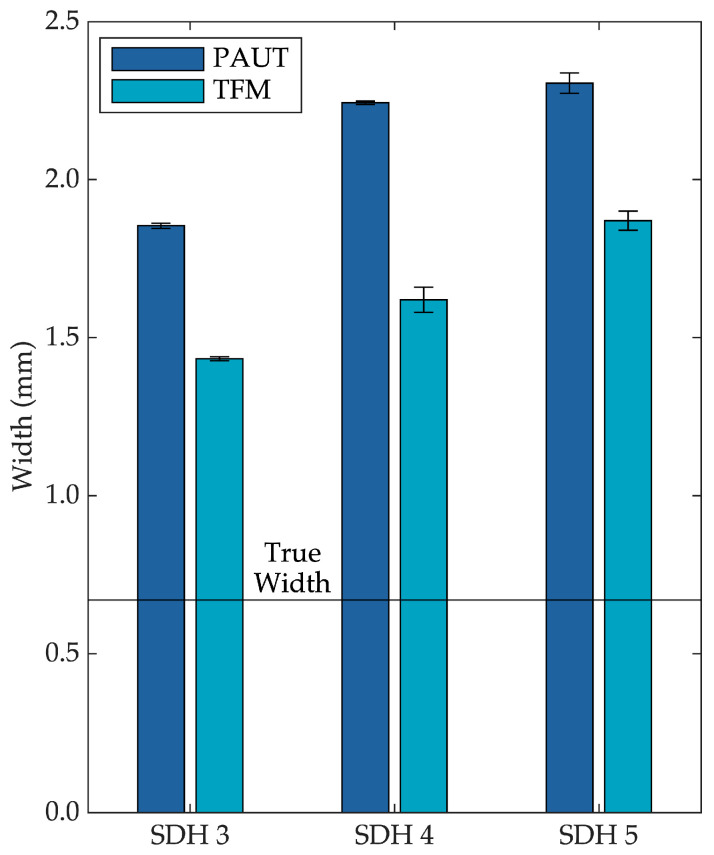
Comparison of flaw sizing performance between PAUT and TFM.

A quantitative comparison of the measured flaw widths is presented in the accompanying bar graph (Figure 11), where each measured width is compared with the actual diameter of the SDH. The results clearly show that PAUT consistently overestimated flaw width to a greater extent than TFM, particularly at larger probe-to-flaw distances. This oversizing trend is attributed to the sectorial beam’s limited focusing capability at off-center positions and its increased angular spread with depth. The operating wavelength in this study was 0.54 mm, corresponding to an approximate axial resolution of ~0.27 mm, which defines the smallest flaw separation that can be reliably distinguished.

In contrast, TFM exhibits more localized and confined flaw indications even at oblique angles, owing to its ability to synthetically focus on every point within the region of interest. As a result, TFM provides more consistent and accurate sizing across different probe positions, particularly when the flaw is located farther from the central beam path. These findings demonstrate the robustness of TFM in maintaining sizing accuracy under varying probe positions and highlight its advantage over conventional PAUT for high-precision flaw characterization.

## 4. Discussion

This study evaluated and compared the performance of PAUT and FMC-based TFM for detecting and sizing near-surface flaws in an additively manufactured Ti-5553 reference block. Beyond comparing the two techniques, this study contributes a controlled experimental dataset for shallow-flaw detection and sizing in AM titanium, providing a reproducible reference benchmark for future optimization and validation of ultrasonic inspection methods. Both techniques successfully detected side-drilled holes with a diameter of 0.67 ± 0.05 mm at depths ≥1.25 ± 0.05 mm using longitudinal waves at normal incidence. Increasing the inspection angle improved detection due to greater separation between front-wall and flaw echoes. TFM demonstrated superior near-surface sensitivity, clearly detecting flaws as shallow as 0.40 ± 0.05 mm at inspection angles of 15° or greater, whereas PAUT’s minimum detectable depth was 1.25 ± 0.05 mm at 0°, improving to 0.90 ± 0.05 mm at 30° refracted angle. This highlights the value of TFM, as it mitigates front-wall interference and provides more reliable detection of near-surface flaws that conventional ultrasonic methods often fail to resolve effectively.

PAUT employs simultaneous excitation of multiple probe elements to generate a focused acoustic beam through constructive interference. This beamforming strategy delivers high acoustic energy to the inspection region, producing strong reflected signals with high signal-to-noise ratios. By contrast, FMC sequentially activates individual elements and records all transmit-receive combinations, distributing energy more broadly and resulting in lower acoustic intensity per transmission. Consequently, while PAUT typically produces stronger echo amplitudes, FMC data provide greater flexibility in post-processing, enabling advanced reconstruction methods such as TFM. The differences in wavefront structure between these methods were evident in energy-scaled wavefield images, where PAUT displayed concentrated energy beams and FMC exhibited more diffuse profiles.

The trade-offs between PAUT and TFM were evident in both detection and sizing performance. PAUT’s high-energy focus enables strong reflectivity and efficient flaw detection. However, its performance degrades at larger probe-to-flaw offsets due to beam divergence, steering-induced distortion, and reduced angular resolution. TFM, although limited by acquisition time and computational overhead, provides superior image clarity, flaw localization, and consistent detection performance, particularly for near-surface flaws.

Accurate flaw sizing is crucial for structural integrity assessments, and the resolution of the ultrasonic image plays a central role in this process. In this study, flaw dimensions were estimated using amplitude drop-based segmentation in both PAUT and TFM images. While PAUT’s sectorial S-scan enhances angular coverage, it was constrained by hardware limitations such as the minimum steering step size and increased beam spread with depth. These factors contributed to oversizing errors, particularly at off-center probe positions.

In contrast, TFM reconstructs images on a defined Cartesian grid, with resolution controlled by the grid spacing. Finer spacing yields sharper flaw boundaries and improved sizing accuracy, although it increases processing demands. In this study, acquisition times for PAUT and FMC were identical, since both datasets were captured simultaneously using the OEM-PA system. The key difference lay in reconstruction: PAUT images were generated in real time, whereas TFM required several seconds per frame in MATLAB. With optimized commercial systems, TFM processing can approach nearly real time. Despite this trade-off, TFM consistently provided more accurate and narrower flaw indications across all probe positions. By synthetically focusing at every point within the region of interest, TFM enabled high-resolution localization and reduced the sizing errors commonly observed with PAUT. These findings confirmed the effectiveness of TFM for precise flaw characterization, particularly when detecting shallow flaws that are difficult to distinguish from front-wall echoes.

As detailed in Section 3.4.3, PAUT exhibited increased oversizing errors with probe offset, primarily due to beam divergence and reduced angular resolution. Although a 10 MHz transducer theoretically enables sub-millimeter resolution, factors such as attenuation and microstructural noise limit its performance. Under these conditions, TFM provided more consistent sizing, confirming its robustness for flaw characterization.

However, widening of flaw indications was observed in TFM images at larger probe offsets due to beam divergence. This effect could be mitigated through post-processing techniques, such as point spread function based deconvolution, which models the system response to a point reflector and corrects for geometric spreading [50]. While this method was not implemented in the present study, it represents a viable direction for enhancing flaw definition in FMC-based systems.

The enhanced performance of TFM in this study was attributed to its fundamental imaging principles, including reduced energy concentration at the front-wall and synthetic focusing at all points within the region of interest. These features help minimize front-wall interference and provide clearer flaw indications, particularly in near-surface regions. Nonetheless, the reliability of TFM depends on careful control of several parameters, including equipment setup, signal quality, and optimal wave mode selection—factors that are often underreported but are critical for achieving consistent and accurate results.

Looking ahead, expanding TFM capabilities through targeted optimization and multi-mode imaging offers considerable potential. While this study focused on a direct comparison of PAUT and TFM under identical acquisition and reconstruction settings, further improvements in TFM are possible through optimizing grid step size for sharper reconstruction and incorporating multi-mode imaging (e.g., 2L, 2T, 3L). Incorporating shear wave modes and higher-order longitudinal reflections could improve volumetric coverage and enhance sensitivity to flaws with varying geometries and orientations. Although these modes were not investigated in the present study due to hardware and software development kit limitations, they represent an important direction for improving inspection reliability in complex AM structures. In addition, future work will focus on combining experimental PSF extraction with advanced simulation approaches (e.g., CIVA or finite-element modelling) to establish absolute sizing accuracy in AM titanium. This will also allow more realistic assessment of the anisotropic microstructure and scattering effects, providing a stronger basis for POD and fitness-for-service (FFS) evaluations.

This study demonstrated that both PAUT and TFM are effective for flaw detection in AM Ti-5553 components. However, TFM provided a substantial advantage in sizing accuracy, near-surface sensitivity, and consistency under variable probe positions. With the growing use of AM in safety-critical applications, demand for advanced imaging techniques such as TFM is expected to increase. Continued progress in post-processing algorithms, wave mode optimization, and scanning strategies will further strengthen the role of TFM in high-precision non-destructive testing of advanced manufacturing components.

While this study focused on 2D reconstructions to enable a controlled comparison between PAUT and TFM, the FMC dataset inherently permits volumetric analysis. Three-dimensional sizing can be achieved by stacking multiple TFM slices, merging data from opposite probe orientations, and rendering the resulting volume to estimate flaw length, width, and depth in a single framework [54]. In parallel, X-ray computed tomography (XCT) provides a complementary 3D reference for validating ultrasonic flaw sizing [55]. Although implementing such 3D analysis was beyond the scope of this study, future work will explore volumetric TFM and XCT reference scans to provide absolute sizing benchmarks. These approaches are consistent with current AM guidance (ISO/ASTM TR 52905 [56]) and evolving FMC/TFM standards (ISO 23865 [16]; ASME Section V [17]), and will support PSF-based accuracy for POD and FFS applications. Collectively, these findings not only clarify the mechanisms behind TFM’s superior near-surface performance, but also establish a validated experimental basis to guide future AM inspection standards and qualification protocols.

## 5. Conclusions

This study demonstrates the effectiveness of the Total Focusing Method (TFM) as an advanced ultrasonic technique for inspecting additively manufactured Ti-5553 components. Building on a controlled comparative dataset of PAUT and TFM inspections in LPBF titanium, this work establishes a reproducible shallow-flaw benchmark (0.40–2.15 mm) that highlights a critical advantage of TFM in resolving near-surface defects. TFM reliably detected side-drilled holes with diameters of 0.67 ± 0.05 mm and depths as shallow as 0.40 ± 0.05 mm, highlighting its capability to resolve shallow flaws at normal incidence.

Compared to PAUT, TFM provided superior image clarity, improved flaw sizing accuracy, and reduced oversizing effects by approximately 1.3 times. Even under AM-specific challenges such as surface roughness and microstructural heterogeneity, TFM consistently produced sharper flaw indications. The use of the longitudinal-longitudinal (L-L) wave mode in TFM imaging further contributed to high-resolution flaw detection, while minimizing signal degradation from front-wall clutter and back-wall reflections.

The MATLAB-based TFM implementation enabled flexible reconstruction with high spatial resolution and offers potential for further enhancement using larger arrays or custom probe geometries. These findings demonstrate that TFM is well suited for near-surface and subsurface inspection of additively manufactured components, offering enhanced sensitivity, improved sizing reliability, and strong compatibility with inspection challenges specific to AM.

Although this analysis was limited to 2D reconstructions for a controlled comparison between PAUT and TFM, future work will extend toward volumetric TFM imaging and complementary XCT validation. Overall, this work supports the advancement of TFM as a preferred technique for accurate flaw sizing in AM structures. Its capabilities make it a highly advantageous tool for non-destructive testing (NDT) applications, where precise flaw detection and characterization are essential.

Beyond these findings, the results also provide practical input for ongoing standardization efforts. In particular, they align with and can inform updates to ISO 23865 [16] (Clauses 8.6, 10.3, and 13.2) and ASME BPVC Section V, Appendix XI (Clauses XI-421, XI-434/462, and XI-451), emphasizing the need to report reconstruction parameters such as grid density when qualifying ultrasonic procedures for AM materials. The study’s outcomes also complement ISO/ASTM TR 52905 (2023) [56], which references PAUT-FMC/TFM trials but remains non-prescriptive regarding TFM parameters or PSF-based corrections. Additional datasets—including flat-bottomed holes, porosity, and lack-of-fusion reflectors—together with PSF validation, are still required to support comprehensive qualification and future updates of AM inspection procedures.

### Industry Implications

The application of TFM significantly enhances the non-destructive testing of additively manufactured components, particularly by improving the detection of near-surface flaws. This capability is essential for qualifying AM parts used in safety-critical industries such as aerospace, medical, and energy sectors, where undetected flaws can lead to premature failure. Moreover, the improved flaw sizing accuracy offered by TFM supports more precise fatigue-life predictions, enabling better-informed maintenance schedules and increasing overall structural reliability. These results may help inform future updates to NDT procedures and standards for quality assurance in safety-critical additive manufacturing applications.

## Figures and Tables

**Table 1:** Dimension and subsurface depth for each side-drilled hole.

SDH ID	1	2	3	4	5
Depth of SDH ± 0.05 (mm)	0.40	0.90	1.25	1.75	2.15

**Table 2:** Key specifications of the ultrasonic phased array transducer used in this study.

Parameter	Value	Unit
Center Frequency	10	MHz
Element width (e)	0.35	mm
Kerf (g)	0.05	mm
Pitch (p)	0.40	mm
Passive aperture (W)	6.35	mm
Bandwidth at −6 dB	78.9	%

**Table 3:** Summary of ultrasonic inspection equipment and instrumentation used, including transducer, acquisition system, and software platforms.

Component	Specification	Manufacturer/Provider
Additive Manufacturing Material	Ti-5553 (LPBF) titanium alloy	Safran Landing Systems Inc.
Reference Block Dimensions	30 mm × 30 mm × 190 mm	Fabricated by Multi-Scale AM Lab, University of Waterloo
Transducer	16-element linear array, 10 MHz, 0.40 mm pitch	Sensor Networks Inc.
Delay Line	15 mm acrylic	Custom-made
Acquisition System	OEM-PA 16/16 phased array unit	Advanced OEM Solutions (AOS)
Software Environment	MATLAB R2022b with in-house TFM algorithm	Custom implementation

**Table 4:** Summary of detection performance of PAUT and TFM techniques for SDHs.

SDH	Depth ± 0.05 mm	PAUT	TFM
1	0.40	✗	✓
2	0.90	✓	✓
3	1.25	✓	✓
4	1.75	✓	✓
5	2.15	✓	✓

(✓ = detected; ✗ = not detected).

**Table 5:** Oversizing factors for 0.67 mm diameter SDH widths measured using PAUT and TFM (*n* = 20), Values are mean [95% confidence interval].

SDH	Depth ± 0.05 mm	Measured Width (mm)		Oversizing Factor	
		PAUT	TFM	PAUT	TFM
3	1.25	1.85 [1.845, 1.855]	1.43 [1.425, 1.435]	2.77 [2.765, 2.775]	2.14 [2.135, 2.145]
4	1.75	2.24 [2.235, 2.245]	1.62 [1.601, 1.639]	3.35 [3.345, 3.355]	2.42 [2.392, 2.448]
5	2.15	2.30 [2.286, 2.314]	1.87 [1.856, 1.884]	3.44 [3.417, 3.463]	2.79 [2.771, 2.809]

## Data Availability

Data is contained within the article.
